# Molecular stratification by BCL2A1 and AIM2 provides additional prognostic value in penile squamous cell carcinoma

**DOI:** 10.7150/thno.51725

**Published:** 2021-01-01

**Authors:** Xingliang Tan, Dong Chen, Shengjie Guo, Yanjun Wang, Yuantao Zou, Zhiming Wu, Fangjian Zhou, Zike Qin, Zhuowei Liu, Yun Cao, Chunhua Lin, Gangjun Yuan, Kai Yao

**Affiliations:** 1 Department of Urology, Sun Yat-sen University Cancer Center, Guangzhou 510060, China.; 2 State Key Laboratory of Oncology in Southern China, Guangzhou 510060, China.; 3 Collaborative Innovation Center of Cancer Medicine, Guangzhou 510060, China.; 4 Department of Pathology, Sun Yat-Sen University Cancer Center, Guangzhou 510060, China.; 5 Department of Urology, The Affiliated Yantai Yuhuangding Hospital of Qingdao University, Yantai, Shandong 264000, China.; 6 Department of Urology Oncological Surgery, Chongqing University Cancer Hospital, Chongqing, 400030, China.

**Keywords:** penile squamous cell carcinoma, comprehensive genomic profiling, BCL2A1, AIM2, molecular classifier

## Abstract

**Background**: Lymph node metastasis is the most unfavorable prognostic factor of penile squamous cell carcinoma (PSCC). However, patients with the same lymph node status have different outcomes, and molecular classifiers for precise prognostic assessments are lacking.

**Methods:** Comprehensive genomic profiling and high-content proliferation screening were performed in eight PSCC and normal tissue pairs and in cell lines. BCL2A1 and AIM2 were selected and further evaluated by qPCR and Western blot. The clinical relevance and prognostic value of the target genes were validated *via* immunohistochemistry in a cohort of 220 PSCC patients with a defined pN stage. Finally, the biological functions and molecular mechanisms of BCL2A1 and AIM2 were investigated *in vitro* and* in vivo*.

**Results:** BCL2A1 and AIM2 were both upregulated in PSCC tissues and associated mostly with cell proliferation. Staining for either BCL2A1 or AIM2 revealed that both are correlated with pN status, extranodal extension, clinical stage and cancer-specific survival (CSS). Compared to patients who are single-positive or double-negative for BCL2A1 and AIM2, those overexpressing both genes had a higher risk of tumor progression and the poorest survival in the pN0 (5-year CSS: 63.3% *vs.* 94.9% and 100.0%, respectively, *p* = 0.000) and pN+ subsets (5-year CSS: 24.1% *vs.* 45.7% and 55.1%, respectively, *p* = 0.035). Molecular biofunction and mechanistic studies demonstrated that BCL2A1 and AIM2 knockdown inhibited tumorigenesis *via* the AIM2/NF-κB/BCL2A1/MAPK/c-Myc signaling pathway.

**Conclusions:** BCL2A1 and AIM2 promote PSCC progression. Integrating BCL2A1 and AIM2 as novel molecular classifiers with pN stage provides additional information for the prognosis and treatment of PSCC patients.

## Introduction

Penile squamous cell carcinoma (PSCC) is a rare but devastating malignancy in males, with an overall incidence of 1/100000 worldwide 1, 2. Compared with the morphological or microscopic patterns of the primary tumor, the presence and extent of lymph node metastasis are the most unfavorable prognostic factors for long-term survival 3-5. The 5-year cancer-specific survival (CSS) rate of patients with pathological node-negative (pN0) PSCC is up to 85% 6, while it decreases dramatically to 29-59% in pathological node-positive (pN+) patients 7-10. However, even in patients with the same lymph node status and histological subtype, the prognosis of individuals varies from person to person due to tumor heterogeneity, as observed in breast or endometrial cancer 11, 12. To complement tumor evaluation precisely, it has become recognized that developments in molecular stratifications provide additional prognostic value and targeted treatment strategies to the classic staging system 13, 14. Hence, imminent work is on the horizon to explore the genomic landscape of PSCC and to ameliorate clinical prognostic stratification with the available molecular subtypes.

Recently, a series of somatic genomic alterations have been identified during the progression of PSCC using a comprehensive genomic profiling (CGP) analysis, which indicated the high mutation burden and molecular complexity of individuals 15-19. However, previous studies were limited to non-whole-transcriptome sequencing 15-17, 19, unpaired PSCC tissues 15, 16, 18, and a lack of large-scale clinical validation and appropriate PSCC cell lines for cellular validation 15-19. Thus, the prognostic value of driver genes is inexplicit, and molecular stratifications of PSCC have not yet been routinely incorporated into clinical practice 1.

In this study, we conducted whole-transcriptome CGP analysis on paired PSCC samples (including primary carcinoma [PCA], metastatic lymph node [LM] and adjacent normal [N] tissues from the same patient) and found that BCL2A1 and AIM2 were upregulated in PSCC and associated with poor survival in a large cohort of 220 PSCC patients with a definite pN stage. The oncogene functions and intrinsic molecular mechanism of BCL2A1 and AIM2 were explored in our newly established PSCC cell lines and animal models 20. We showed that integrating BCL2A1 and AIM2 into a new molecular classifier with a conventional pN status could provide additional prognostic value for subsequent individualized treatments.

## Methods

### Patients and research ethics

This study was approved by the SYSUCC Ethics Committee (No. 2020-FXY-056), and informed consent was acquired (B2020-073). In brief, a total of 220 paraffin-embedded tumor sections (4-µm thickness) and 99 frozen specimens (78 tumor tissues and 21 normal tissues) from 220 patients who were diagnosed with PSCC at Sun Yat-sen University Cancer Center (SYSUCC) between 2000 and 2018 were retrieved. All specimens were redetermined by two pathologists (Keming Chen and Lilin Liu) according to the TNM Staging System for Penile Cancer (8th ed., 2017).

### CGP of invasive PSCC

Next-generation whole-transcriptome CGP was performed on 12 paired PSCC fresh frozen samples (including PCA, LM and corresponding N tissues for each pair). All samples were pathologically confirmed, and total RNA was extracted with TRIzol™ reagent (Invitrogen). RNA quality control criteria were assessed as an RNA integrity number ≥ 7.0; 28S/18S > 0.7 (Agilent 2100 Bioanalyzer) and 1.7 < OD_260/280_ < 2.2 (Thermo NanoDrop 2000) 21. Overall, 24 eligible paired samples (8/12 patients) were subjected to CGP with Affymetrix Microarrays (Shanghai Genechem Co., Ltd.). Gene expression levels were validated and normalized as previously described 22, 23. Then, differentially expressed genes were detected by the Benjamini-Hochberg method 24, and those with a fold change |FC| ≥ 2 are shown. HCPS analysis was subsequently performed with the overlapping overexpressed genes.

### High-content proliferation screening (HCPS)

The shRNA-SET^TM^ lentiviral library (Shanghai Genechem Co., Ltd.) was used to silence the expression of target genes. The criteria of genes eligible for HCPS analysis were as follows: (1) co-upregulated genes that were highly expressed in the LM group; (2) a contained lentiviral library; (3) participation in proliferation- and metastasis-related signaling pathways; and (4) oncogenes reported in human malignancy. In brief, RNA interference plasmids (GV248; hU6-MCS-Ubiquitin-EGFP-IRES-puromycin) were transfected into 149rca PSCC cells and seeded into 96-well plates (1500 cells / well). The cell number was counted for 5 consecutive days and compared with the negative control (FC ≥ 1.5 was significant) to explore the proliferation potential of target genes in PSCC progression.

### Immunohistochemistry (IHC) assay

Paraffin-embedded tissue sections were processed according to standard pathologic procedures. BCL2A1 staining scores were multiplied by the staining intensity (0 for no staining, 1 for weak, 2 for clear and 3 for strong) and staining average (1 for 1-20%, 2 for 20-50%, and 3 for 50% above). The AIM2 IHC score was based on the staining intensity (1 for no staining, 2 for faint and weaker than interstitial cells, 3 for clear and similar to interstitial cells, 4 for yellow-brown and stronger than interstitial cells and 5 for extremely strong). The antibodies and dilutions were as follows: AIM2 (ab93015, Abcam, 1:250) and BCL2A1 (CY5582, Abways, 1:100). The cut-off value was calculated using X-Tile software (version 3.6.1) as previously described 25. In our cohorts, the cut-off values of BCL2A1 and AIM2 IHC scores were both 2 points. A BCL2A1 expression score of 3-9 points and an AIM2 expression score of 3-5 points were regarded as high expression.

### Cell lines, culture conditions and transfection

The Penl2, 149rca and 156lm PSCC cell lines were obtained from our laboratory as previously described 20. The human epidermis keratinocyte cell line (HaCaT), mouse head and neck squamous cell carcinoma (SCC-7) and mouse lung squamous cell carcinoma (KLN205) were obtained from the Type Culture Collection of the Chinese Academy of Sciences (Shanghai, China). Most of the cell lines (KLN205 cells were cultured in Roswell Park Memorial Institute 1640 medium) were cultured in Dulbecco's Modified Eagle's Medium (DMEM) supplemented with 10% fetal bovine serum (Gibco) at 37 °C in a 5% CO_2_ incubator. Full-length BCL2A1 was subcloned into the pIRES2 - EGFP vector (using EcoRI and BamHI) for overexpression plasmids. The lentivirus used for RNA interference was obtained from the shRNA-SET^TM^ lentiviral library (Shanghai Genechem Co., Ltd.). The effective shRNA sequences were as follows: BCL2A1-sh1, GCCAGAACACTATTCAACCAA; BCL2A1-sh2, TGCGTCCTACAGATACCACAA; AIM2-sh1, AAGGGTTTCAGAAGCGCTGTT; AIM2-sh3, CAGCTGACATCTGGAGTTCAT; and shNC, TTCTCCGAACGTGTCACGT.

### Quantitative real-time PCR assay

Total RNA was extracted with a HiPure Total RNA Plus Micro Kit (Magen). HiScript Q RT SuperMix (Vazyme) and ChamQ SYBR qPCR Green Master Mix (Vazyme) were used for cDNA synthesis and amplification according to the manuals. Primers for the corresponding target genes were as follows: GAPDH forward 5'-TGGTGAAGACGCCAGTGGA-3' and reverse 5'-GCACCGTCAAGGCTGAGAAC-3'; BCL2A1 forward 5'-TACAGGCTGGCTCAGGACTAT-3' and reverse 5'-CGCAACATTTTGTAGCACTCTG-3'; and AIM2 forward 5'-TGGCAAAACGTCTTCAGGAGG-3' and reverse 5'-AGCTTGACTTAGTGGCTTTGG-3'.

### Western blot (WB)

Cells were lysed in RIPA lysis buffer (Beyotime) containing 1% phosphatase and protease inhibitors at 4 °C for approximately 30 min. Protein (30 μg) was separated by 10% SDS-PAGE (PAGE-Gel Fast Preparation Kit, EpiZyme) and transferred onto PVDF membranes (Pierce Biotechnology). Protein bands were blocked with 5% nonfat milk at room temperature for 2 h and incubated with the indicated primary antibody overnight at 4 °C and the appropriate secondary antibody for 2 h. ECL reagents (Abcam) were used for exposure. The following antibodies and dilutions were used: AIM2 (ab93015, Abcam, 1:1000), BCL2A1 (CY5582, Abways, 1:1000), p44/42 MAPK (137F5, CST, 1:1000), c-Myc (E5Q6W, CST, 1:1000), α-tubulin (AF0001, Beyotime, 1:1000), NF-κB Pathway Sampler Kit (#9936, CST, 1:1000), Phospho-Erk1/2 Pathway Sampler Kit (#9911, CST, 1:1000), IL-1β (#12703, CST, 1:1000), cleaved IL-1β (#83186, CST, 1:1000) and Caspase-1 (#3866, CST, 1:1000).

### IL-1β and IL-18 ELISAs

The concentrations of IL-1β and IL-18 were determined using the corresponding enzyme-linked immunosorbent assay (ELISA) kit (Telenbiotech, TL-E083 and TL-E092) according to the manufacturer's instructions. Briefly, 5×10^6^ Penl2 and 149rca PSCC cells were seeded in 60-mm dishes. After the cells were allowed to attach for 24 hours, the medium was changed, supplemented with/without 5 μg/ml lipopolysaccharide (LPS), and incubated with the cells for 4 h to activate the immune response. Then, supernatants were collected, and the levels of secreted inflammatory cytokines in the culture medium were measured in triplicate.

### *In vitro* tumorigenesis assays

Cell proliferation, migration, colony formation and wound healing assays were performed to evaluate the biological functions of the target genes. In brief, for cell proliferation, PSCC cells (2×10^3^/well) were seeded into 96-well plates with 100 μl medium, mixed with 10 μL CCK-8 solution (Dojindo, Japan) and incubated for 2 h. The cell number was counted by the optical density (OD) value at an optimal wavelength of 450 nm on an Infinite F50 microplate reader for 7 consecutive days. For cell migration, 10^5^ cells were cultured in 300 μl serum-free DMEM and seeded into the upper compartment of a 24-well transwell culture chamber, and 750 μl DMEM supplemented with 10% serum was added to the lower compartment. Migrated cells were counted after incubation for 24 h. For colony formation, PSCC cells (1×10^3^/well) in 6-well plates were cultured for 10 days and fixed with 4% paraformaldehyde for 30 min. The number of colonies was counted by ImageJ (National Institutes of Health, USA) after staining with 0.5% crystal violet. For wound healing, cells were seeded into 6-well plates after growing to confluence, and cross wound lines were made with a 1000-μl pipette tip. Then, the cells were cultured in serum-free DMEM for 16 h, and the wound healing area was recorded. All the experiments were repeated in triplicate.

### *In vivo* nude mice tumorigenesis model and syngeneic tumor model

The animal experiments were approved by the SYSUCC Animal Ethics Committee (L102022019001I). All mice were purchased from Jiangsu GemPharmatech Co., Ltd. For the nude mice tumorigenesis model, six- to eight-week-old male BALB/c nude mice were randomly divided into three groups (n = 6 per group) with similar weights and maintained under the same feeding conditions. A suspension of 10^6^ shBCL2A1, shAIM2 or shNC-Penl2 cells in 150 μl of saline was subcutaneously injected into the right flank of each nude mouse (n = 6 mice per group). The tumor volume and weight of mice were monitored twice a week by the same observer. Mice were euthanized when weight loss was over 20% or when the tumor size was greater than 1500 mm^3^. Three weeks after tumor formation, tumors were retrieved and weighed. Tumor volume was calculated as follows: Volume (mm^3^) = 0.5×length×width^2^. Similarly, for the syngeneic tumor model, six- to eight-week-old male C57/BL6 mice (n = 5 per group) was subcutaneously injected with 10^6^ SCC-7 or KLN205 cells transfected with shAIM2 or shNC, and mice were sacrificed two weeks later to measure the tumor volumes and weights.

### Statistical analysis

Statistical analysis was performed with SPSS software (Version 25.0) and GraphPad Prism (Version 7.00). Statistics are presented as the means ± SDs of three independent experiments and were analyzed by Student's *t*-test or one-way ANOVA. Two or more sample composition ratios were determined by the chi-square test. Survival analysis was based on the Kaplan-Meier survival curve and multivariate Cox proportional hazards regression model by the forward method. Fisher's exact test was used for small samples, and a *p*-value < 0.05 was considered significant.

## Results

### Genomic landscape of eight pairs of PSCC tissues and HCPS results

**Figure 1:**
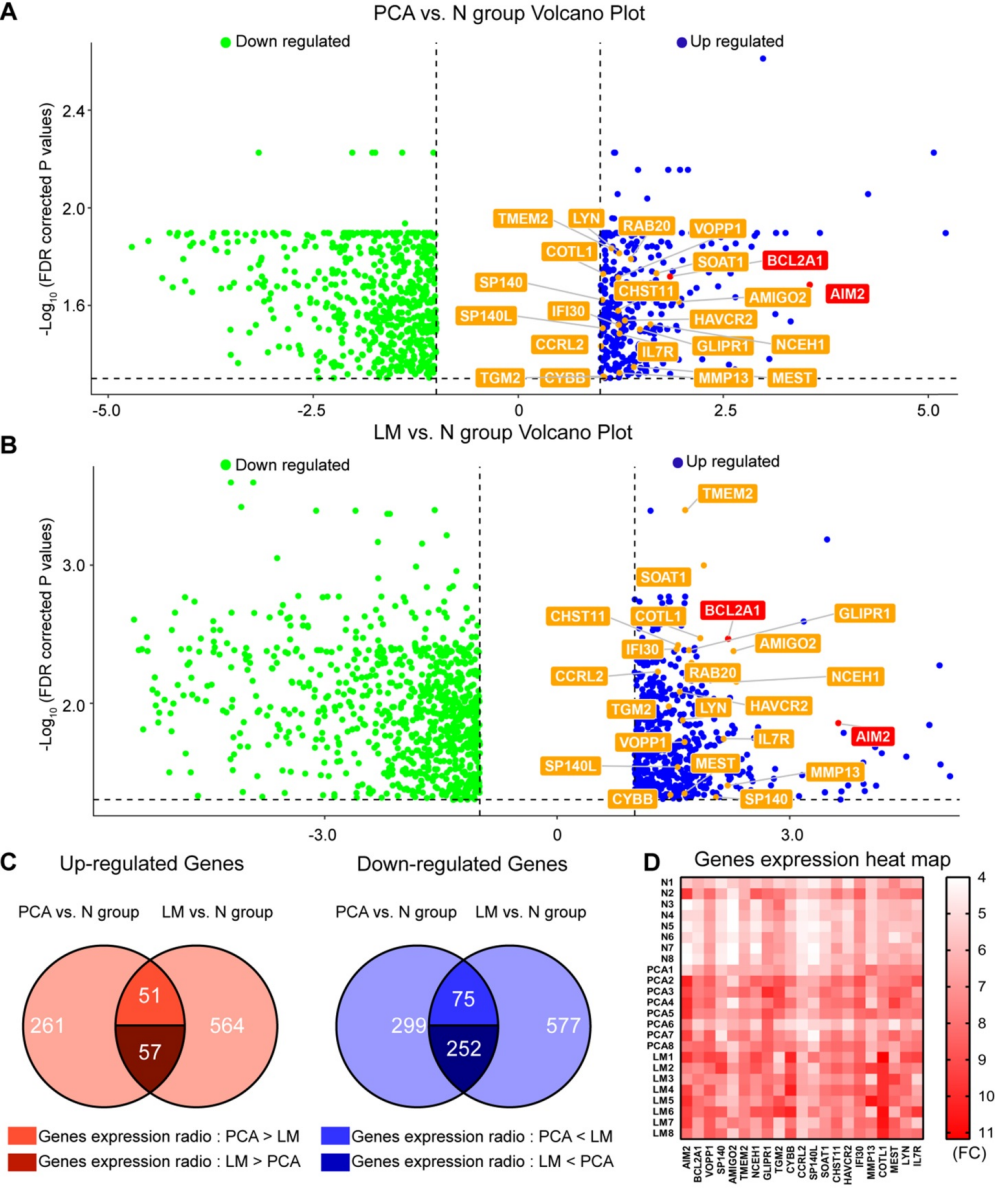
** Gene expression pattern of eight invasive PSCC patients.** (**A**) Venn diagram showing 369 and 672 overexpressed genes in the PCA group and LM group, respectively, and among the 108 co-upregulated genes, 57 were more highly expressed in the LM group and 51 were more highly expressed in the PCA group. Furthermore, 327 co-downregulated genes were identified in the two groups, of which 252 had lower expression in the LM group. (**B**) Gene expression heat map indicated that 22 target genes were overexpressed in the LM and PCA groups; these genes were subjected to subsequent HCPS analysis. (**C**) Differentially expressed genes (|FC|>2 and FDR<0.05) in the PCA and LM groups are shown as volcano plots in which 22 co-upregulated genes are labeled. PSCC, penile squamous cell carcinoma; FC, fold change; FDR, false discovery rate; N, adjacent normal; PCA, primary carcinoma; LM, metastatic lymph node.

**Figure 2:**
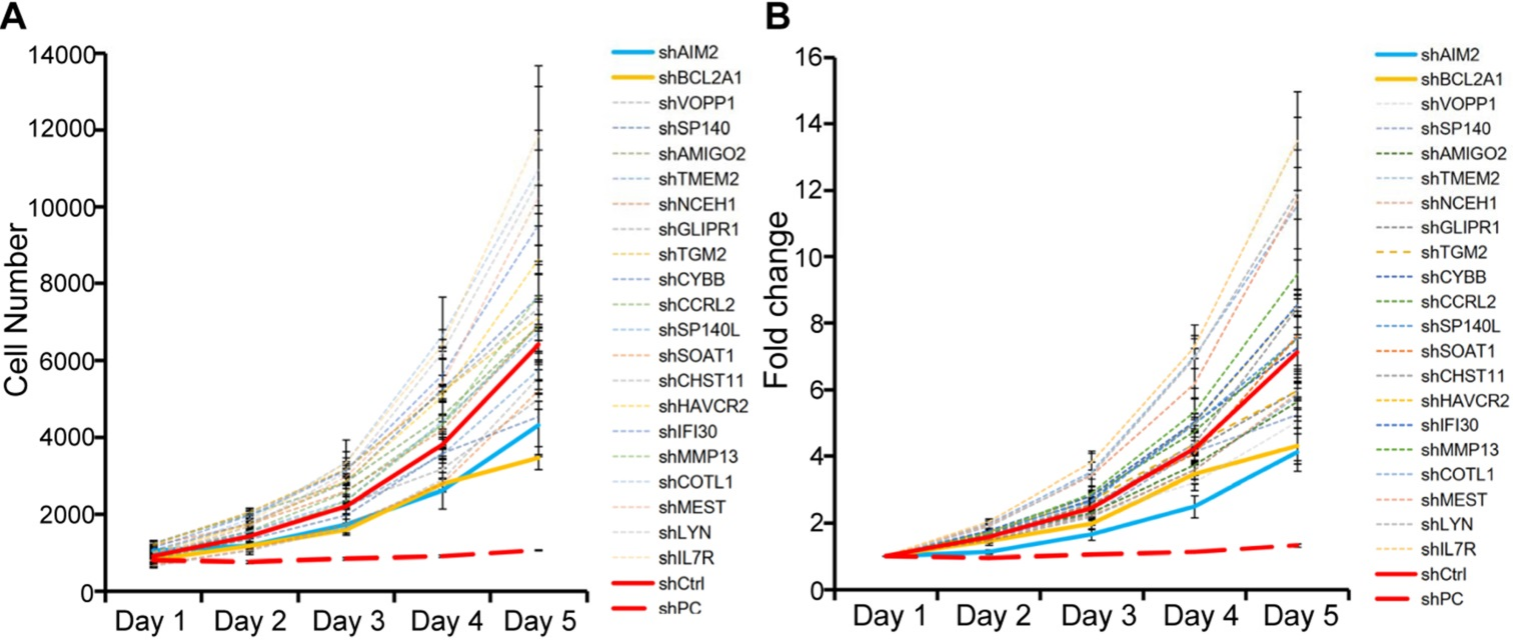
** The HCPS results of 22 co-upregulated genes.** (**A-B**) Cell proliferation curves of HCPS results. Compared with the shCtrl group, the FC values of the shBCL2A1, shAIM2 and shPC groups were 1.65, 1.73 and 5.38, respectively. HCPS, high-content proliferation screening; shPC, positive control; shCtrl, negative control.

To investigate gene heterogeneity and molecular changes in PSCC, PCA, LM and corresponding N tissues from 8 pN+ PSCC patients (Table S1) were collected for whole-transcriptome CGP analysis (Figure 1A-B). Next, overlapping genes that were highly expressed or downregulated in both the PCA and LM groups were selected as shown in Figure 1C, with a total of 57 genes that were significantly co-upregulated and expressed at higher levels in LM than in PCA tissues. Among the 57 genes, 22 that correlated with cancer cell proliferation and metastasis were screened for further HCPS analysis (Figure 1D). Our results suggested that the knockdown of BCL2A1 or AIM2 in 149rca PSCC cells extremely inhibited cell growth compared with the other 20 genes and the negative control (Figure 2A-B). We speculate that both BCL2A1 and AIM2 are implicated in the tumor proliferation of PSCC.

### BCL2A1 and AIM2 are overexpressed in PSCC tissues and cell lines

**Figure 3:**
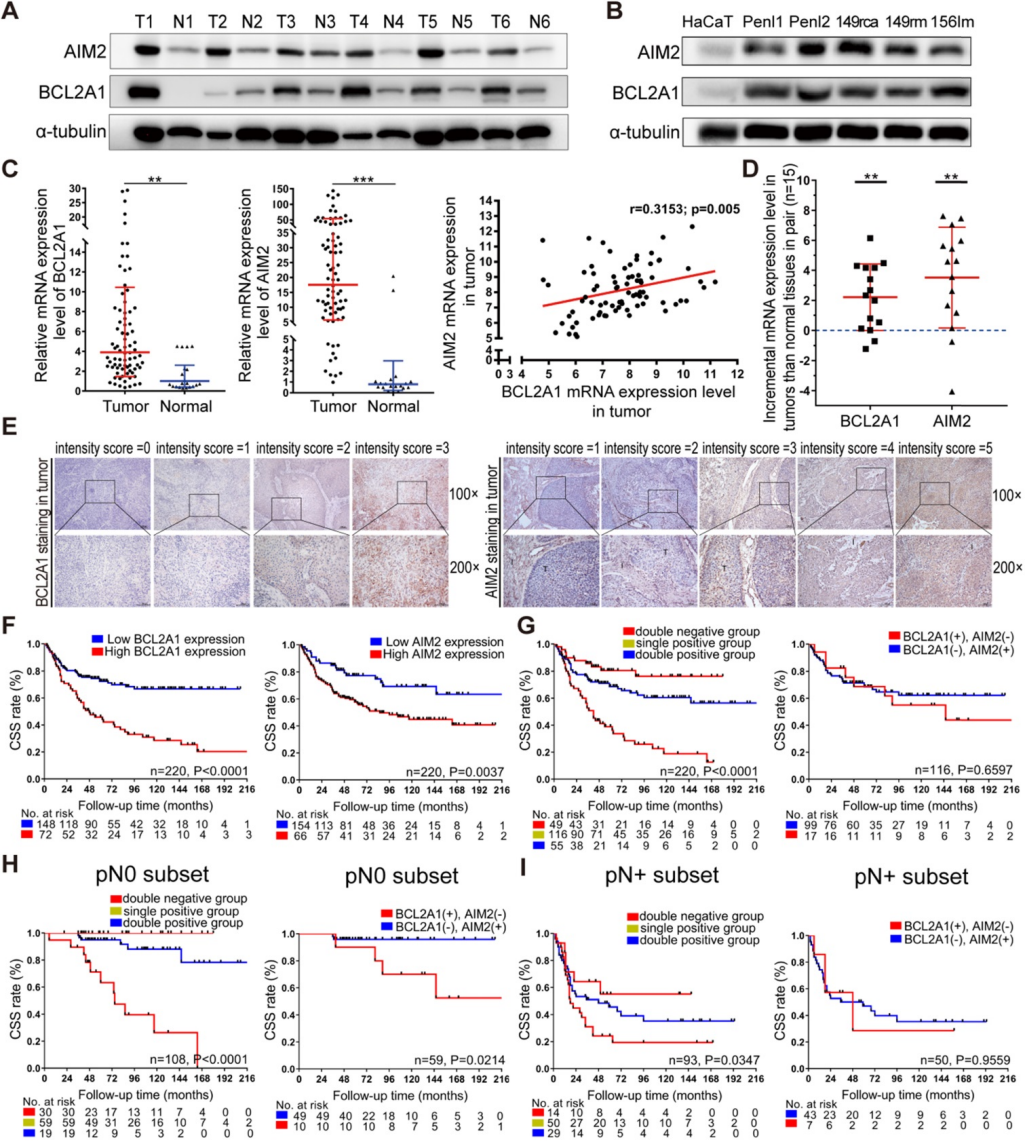
** Expression levels of BCL2A1 and AIM2 in PSCC and their correlation with survival.** (**A, B**) The BCL2A1 and AIM2 proteins were overexpressed in PSCC tumors and cell lines compared with the corresponding normal tissues and HaCaT cells. (**C**) The mRNA levels of BCL2A1 and AIM2 with positive expression were upregulated in 78 tumor tissues compared with 21 normal tissues. (**D**) The mRNA expression levels of BCL2A1 and AIM2 were higher in tumor tissues than in paired N tissues (n = 15). The mRNA expression levels are presented as the means ± SDs of three independent experiments. (**E**) The standard staining intensity score of BCL2A1 in the cytoplasm was 0 for no staining, 1 for weak staining, 2 for clear staining and 3 for strong staining. For AIM2, the standard was 1 for no staining in the tumor, 2 for faint staining in the tumor and weaker than in interstitial cells, 3 for clear and similar to that in interstitial cells, 4 for yellow-brown staining stronger than that in interstitial cells and 5 for extremely strong staining in the tumor (T, tumor cells; I, interstitial cells). (**F**) Kaplan‑Meier survival analysis indicated that overexpression of both BCL2A1 and AIM2 was associated with poor CSS in PSCC patients. (**G-I**) Patients with double-positive expression (BCL2A1^+^ AIM2^+^) had a shorter CSS rate than the single-positive group (BCL2A1^-^ AIM2^+^ or BCL2A1^+^ AIM2^-^) and the double-negative group (BCL2A1^-^ AIM2^-^) in the whole cohort as well as in the pN0 or pN+ subset. *p < 0.05, **p < 0.01, ***p < 0.001. IHC, immunohistochemistry; HaCaT, human immortalized keratinocytes; PSCC, penile squamous cell carcinoma.

To validate the expression of BCL2A1 and AIM2 in pN0 PSCC patients, 6 pairs of fresh PSCC and N tissues as well as PSCC cell lines were prepared and monitored by WB assays. The results showed that BCL2A1 and AIM2 proteins were highly expressed in tumor tissues and PSCC cells compared to normal controls (Figure 3A-B). The qPCR assay revealed that the mRNA expression of BCL2A1 and AIM2 was positively correlated (r = 0.315, *p* = 0.005) and significantly overexpressed in 78 PSCC tissues compared with 21 normal tissues (*p* = 0.002; *p* = 0.001) (Figure 3C). Further analysis of the mRNA expression of 15 pairs of PSCC and N tissues showed that BCL2A1 and AIM2 were overexpressed in tumor tissues compared with the corresponding control tissues, consistent with the WB results (Figure 3D).

### Relationship between BCL2A1 or AIM2 expression and clinical features in 220 PSCC patients by IHC

To further explore the association between BCL2A1 or AIM2 expression and clinical features, 220 paraffin-embedded PSCC sections were prepared and subjected to IHC assays. The IHC results are summarized in Table 1. In our cohort, the median follow-up time after surgery was 99.3 months (IQR: 53.0-138.0), and 92 (41.8%) patients died of PSCC. As detected by IHC, both the BCL2A1 and AIM2 proteins were stained in the cytoplasm; the patterns are shown in Figures 3E and S1. The IHC scoring criteria are described in the Materials and methods section, and the frequency in each subgroup is shown in Figure S1. In brief, the BCL2A1 protein was detected in 154 of 220 (70.0%) PSCC patients, 72 of whom (32.7%) had high expression (IHC score >2), while 148 (67.3%) had low expression. Regarding the AIM2 protein, 154/220 (70.0%) patients overexpressed AIM2 (IHC score >2), while 66/220 (30.0%) had low AIM2 expression. Chi-square tests indicated that the high expression of both BCL2A1 and AIM2 was correlated with a poor pN status (*p* = 0.005; *p* = 0.031), clinical stage (*p* = 0.001; *p* = 0.022) and extranodal extension (ENE) (*p* = 0.003; *p* = 0.045). In addition, AIM2 overexpression was associated with the pT status (*p* = 0.016), but BCL2A1 was not (*p* = 0.152) (Table 1).

### Cumulative effect of the overexpression of BCL2A1 and AIM2 on poor outcomes

To determine the relationship between BCL2A1 or AIM2 expression and clinical outcomes, Kaplan-Meier survival analyses were performed. Our results indicated that PSCC patients with BCL2A1 (*p* = 0.000) or AIM2 (*p* = 0.004) overexpression experienced poor CSS (Figure 3F). Interestingly, if we combined the expression statuses of the two genes, CSS was significantly shorter when the number of positive genes increased (Figure 3G). The 5-year CSS rate in the double-positive group (BCL2A1+ AIM2+) was only 38.9%, which was significantly lower than that in the single-positive group (68.6%; BCL2A1+ AIM2- or BCL2A1- AIM2+) and in the double-negative group (80.5%; BCL2A1- AIM2-) (both *p* = 0.000), though no significant difference was detected between the single BCL2A1-positive and single AIM2-positive groups (68.6% *vs.* 68.4%, *p* = 0.660) (Table 2 and Figure 2G). Other clinical features, including the pT stage, pN status, metastasis, pathological grade, clinical stage and ENE, were also related to CSS (both *p*<0.001, Figure S2), consistent with a previous report 7. Moreover, multivariate analysis demonstrated that BCL2A1 expression (*p* = 0.044, HR = 1.691; 95% Cl: 1.014-2.820), pT stage and N status were independent prognostic indicators of CSS for PSCC patients (Table 3). These findings suggest that the overexpression of BCL2A1 and AIM2, with a cumulative effect, is associated with a poor prognosis in PSCC patients.

### BCL2A1 and AIM2 provide additional prognostic value, especially in pN0 PSCC patients

To further demonstrate the prognostic value of BCL2A1 and AIM2 expression in different pN subsets, the patient cohort was stratified into the pN+ (112/220, 50.9%) and pN0 (108/220, 49.1%) groups. In the pN0 subset, the prognosis of patients with double-positive expression was the worst, with a 63.3% 5-year CSS rate, compared to the single-positive group (5-year CSS: 94.9%, *p* = 0.000) and the double-negative group (5-year CSS: 100.0%, *p* = 0.000) (Figure 3H and Table 2), while pT grade (*p* = 0.412) and G grade (*p* = 0.892) were not associated with CSS in pN0 patients (Figure S3A). Among the single-positive group, patients with single BCL2A1 overexpression had poorer CSS than those with single AIM2 overexpression (Figure 3H and Table 2). A similar tendency was also observed in the pN+ subset. When the number of positive genes increased, the prognosis of pN+ patients worsened (*p* = 0.035) (Figure 3I). The 5-year CSS rate of PSCC patients with double-positive expression was significantly lower than that of patients with double-negative expression (24.1% *vs.* 55.1%, *p* = 0.049), but no difference was detected in the remaining groups (Figure 3I and Table 2). In addition, pT grade and G grade (both *p*<0.001) were associated with CSS in the pN+ subset (Figure S3B). Our findings suggest that molecular stratification by BCL2A1 and AIM2 provides additional prognostic value, especially in pN0 PSCC patients.

### Knockdown of BCL2A1 and AIM2 inhibits tumorigenesis *in vitro* and *in vivo*

**Figure 4:**
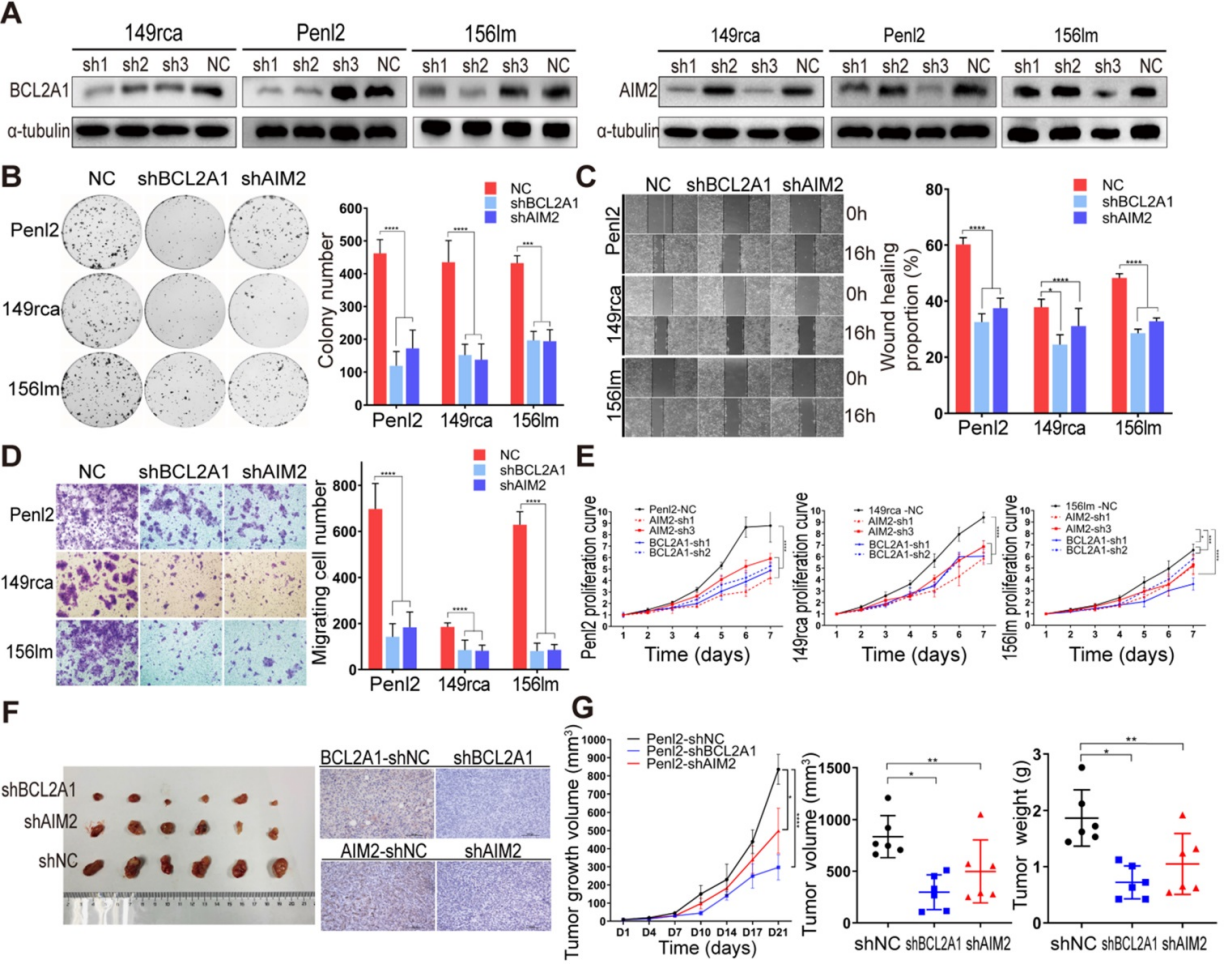
** BCL2A1 and AIM2 knockdown inhibited tumorigenesis *in vitro* and *in vivo*.** (**A**) Knockdown efficiency of levels of the BCL2A1 and AIM2 proteins was detected using WB. The level of the BCL2A1 protein decreased significantly in cells transfected with the BCL2A1-sh1/-sh2 plasmids and AIM2 levels were reduced in cells transfected with the AIM2-sh1/-sh3 plasmids. (**B-D**) Penl2, 149rca and 156lm cells were transfected with equal proportions of BCL2A1-sh1 and -sh2 plasmids or AIM2-sh1 and -sh3 plasmids to knock down the expression of the corresponding gene. BCL2A1- and AIM2-silenced PSCC cells exhibited significantly reduced clone formation (B), wound healing (C) and numbers of migrating cells (D). (**E**) Knockdown of BCL2A1 and AIM2 inhibited PSCC cell proliferation. (**F and G**) Knockdown of BCL2A1 and AIM2 significantly inhibited tumor growth *in vivo*. IHC was performed to confirm the expression levels of BCL2A1 and AIM2 in each group. Statistics are presented as the means ± SDs of three independent experiments. *p < 0.05, **p < 0.01, ***p < 0.001, ****p < 0.0001.

To investigate the biofunctions of BCL2A1 and AIM2 in the progression of PSCC, we first knocked down the protein expression of BCL2A1 and AIM2 in 149rca, Penl2 and 156lm PSCC cells (Figure 4A). Colony formation and CCK-8 proliferation assays revealed that the knockdown of BCL2A1 and AIM2 dramatically impaired cell growth, indicating that BCL2A1 and AIM2 are critical for PSCC cell proliferation (Figure 4B and E). Additionally, the knockdown of BCL2A1 and AIM2 decreased migration, as shown in Figure 4C-D. Moreover, the results of *in vivo* experiments in nude mice showed that the knockdown of BCL2A1 or AIM2 resulted in lighter and smaller tumors, respectively, than the control (Figure 4F-G).

### The AIM2/NF-κB/BCL2A1/MAPK/c-Myc axis is a potential molecular pathway in PSCC progression

**Figure 5:**
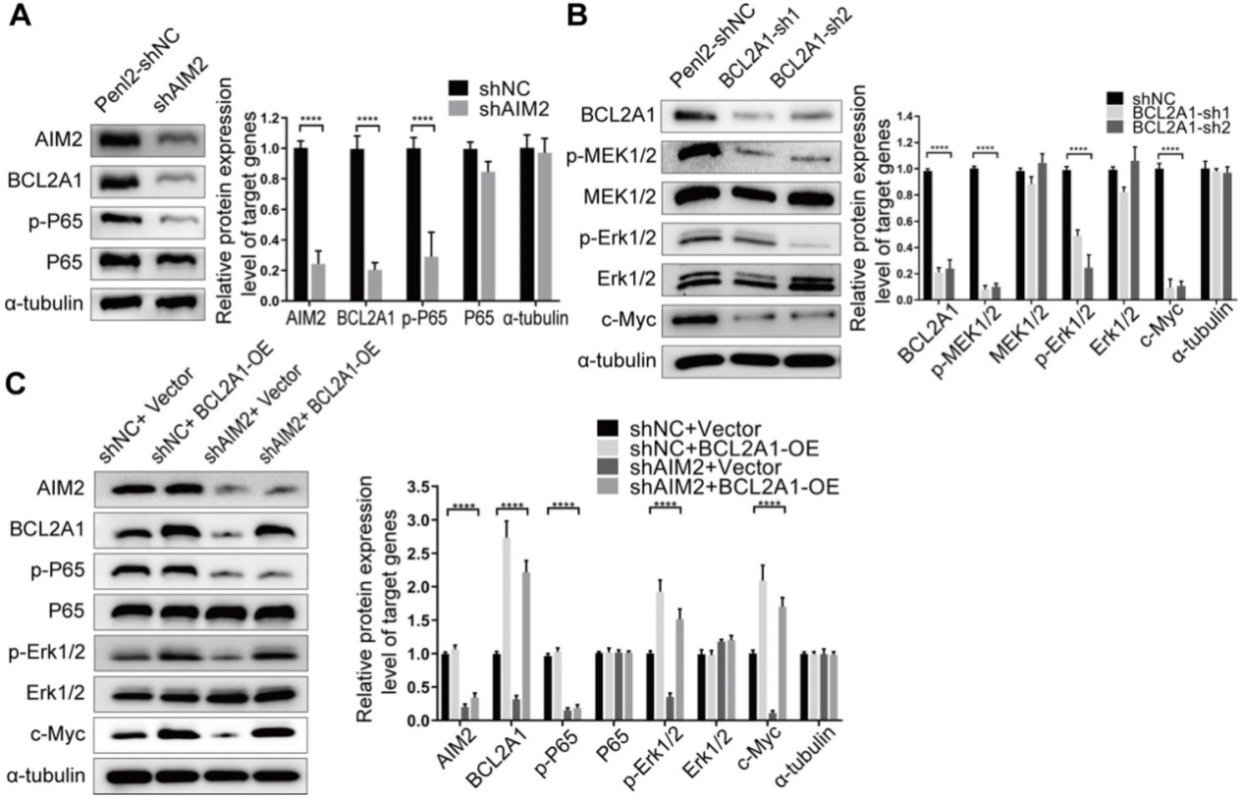
** The AIM2/NF-κB/BCL2A1/MAPK/c-Myc signaling pathway was the potential molecular mechanism for tumor progression in PSCC. (A)** The knockdown of AIM2 protein expression in Penl2 cells reduced the levels of phosphorylated p65 in the NF-κB pathway and the level of the BCL2A1 protein. **(B)** The knockdown of BCL2A1 protein expression in Penl2 cells reduced the levels of phosphorylated of MEK1/2 and Erk1/2 in the MAPK pathway and regulated the effect of c-Myc on cell proliferation. **(C)** The overexpression of shAIM2 in Penl2 cells by transfection with the BCL2A1 plasmid revealed that the NF-κB and MAPK pathways participate in cell proliferation, as evidenced by WB of recovery experiments. Statistics are presented as the means ± SDs of three independent experiments. ****p < 0.0001. NF-κB, nuclear factor kappa-B; MAPK, mitogen-activated protein kinase.

To observe the intrinsic connection between BCL2A1 and AIM2, we first silenced the expression of one gene and detected the expression of the other by WB. The knockdown of AIM2 resulted in a decrease in BCL2A1 expression, whereas the repression of BCL2A1 had no effect on AIM2 expression, indicating that AIM2 is an upstream regulator of BCL2A1 (Figure 5A). It has been reported that the knockdown of AIM2 downregulates nuclear factor κB (NF-κB) expression, resulting in the suppression of cell growth and apoptosis in oral squamous cell carcinoma 26, while the transcription factor NF-κB binds to the BCL2A1 promoter induces its mRNA transcription 27. In addition, the downregulation of BCL2A1 inhibits cell proliferation and metastasis in triple-negative breast cancer by reducing the phosphorylation of ERK1/2 and the expression of c-Myc *via* the MAPK pathway 28. Thus, we wondered whether similar molecular changes occurred in PSCC cells when AIM2 or BCL2A1 was depleted. As expected, knockdown of the AIM2 protein in Penl2 cells significantly decreased the phosphorylation of p65, a dominant protein in the NF-κB subunit family 29, while BCL2A1 silencing reduced the levels of the phosphorylated MEK1/2, Erk1/2 and c-Myc proteins (Figure 5A-B). Recovery experiments indicated that when AIM2-silenced Penl2 cells were transfected with BCL2A1 plasmids, the ratio of p-ERK1/2 and c-Myc proteins was restored without affecting the level of phosphorylated p65 (Figure 5C). These results demonstrate that the AIM2/NF-κB/BCL2A1/MAPK/c-Myc axis is a potential molecular pathway in the progression of PSCC.

### The AIM2 expression of PSCC cells doesn't affect the release of inflammatory cytokines

As shown in previous studies, AIM2, a dominant component of AIM2 inflammasome, plays an important role in programmed cell death by triggering innate immune response 30-32. The AIM2 inflammasomes in immune cells activate Caspase-1 to cleave anti-tumor inflammatory cytokines such as pro-interleukin (IL)-1β and pro-IL-18 into their active forms, inducing apoptosis, pyroptosis and necroptosis 30-32. However, such role of AIM2 in PSCC cells remains blank, so we further detected the expression of inflammatory cytokines by ELISAs and WB. Our results indicated that cleaved IL-1β and IL-18 were extremely low, regardless of the expression level of AIM2 in tumor cells (Figure S4A-B). Knockdown of AIM2 in Penl2 and 149rca PSCC cells reduced Caspase-1 protein, but did not alter cleaved IL-1β levels (Figure S4C). Only when LPS was added exogenously, the AIM2 inflammasome in PSCC cells was activated and higher cleaved IL-1β and IL-18 levels were observed in NC-transfected cells than in the AIM2-silenced cells (Figure S4A-C).

### Knockdown of AIM2 in SCC-7 cells inhibits tumor growth in immune-competent mice

To further investigate whether AIM2 expression in PSCC cells affects tumor growth in immune-competent mice. We established the syngeneic subcutaneous tumor models with SCC-7 and KLN205 cell lines owing to the similar pathological types and oncogenic functions 26, 33. We detected that knockdown of AIM2 in SCC-7 cells resulted in smaller and lighter tumors, while no difference in tumors formed by KLN205 cells was observed compared to the control (Figure S5). The results suggested that AIM2 might have a direct effect on the growth of cancer epithelial cells.

## Discussion

The TNM staging system is a widely used and powerful tool for the prognostic assessment of cancer patients, of which pN stage is the core indicator of survival in PSCC patients 3-5. However, we found in clinical practice and in other studies that there is a certain difference in the prognosis of PSCC patients even with the same pN stage that was attributed to tumor heterogeneity 5-8. Currently, the NCCN guidelines for breast or testicular cancer explicitly propose molecular (ER, PR and HER2) or serological (AFP, β-hCG and LDH) markers as supplements to the TNM stage for a more accurate prognostic assessment 34, 35. However, a precise molecular typing study on the stage of penile cancer has not been conducted 1. Fortunately, rapid advances in next-generation CGP technologies have provided a better understanding of tumor progression and were beneficial for risk assessments, a more precise prognosis and tailored therapy 36, 37. PSCC is a heterogeneous disease, harboring approximately 5.45 genomic alterations per tumor 15. In contrast to other malignancies with a high mutation burden, such as colorectal cancer 13 or muscle-invasive bladder cancer 38, there is no available molecular classifier to accurately assess the prognosis of an individual with PSCC. The underlying reasons could be the lack of suitable paired PSCC tissues and cell lines for comprehensive sequencing and convictive experiments on specific biomarkers 15-19, 39.

To this end, whole-transcriptome CGP analysis was conducted in eight pairs of PSCC tissues, including PCA, LM and N tissues. We found that 22 genes were co-upregulated in PSCC tissues, including BCL2A1 and AIM2, which were associated mostly with cell proliferation *via* HCPS analysis. Consistent with the CGP results, the mRNA expression of BCL2A1 and AIM2 was higher in 78 tumor tissues than in 21 normal tissues. Although tumor heterogeneity is a challenge in the use of genomic-based prognostic markers 40, our results suggest the low intratumor variability of BCL2A1 and AIM2 during tumor progression. Our results also indicate that BCL2A1 and AIM2 are reliable and specific oncogenes in PSCC. BCL2A1 is an antiapoptotic oncogene that prevents cytochrome c translocation from the mitochondria to the cytoplasm and subsequent activation of the intrinsic apoptotic pathway, and it is overexpressed in advanced cancers and correlates with a poor prognosis 27, 28, 41-43. Overexpression of AIM2 has been reported in oral squamous cell carcinoma and non-small cell lung cancer and is associated with shorter survival in patients with oral squamous cell carcinoma 26, 33.

To further reveal the clinical significance of BCL2A1 and AIM2 in PSCC, IHC was conducted in a large cohort of 220 PSCC patients. The results showed that either BCL2A1 or AIM2 staining was significantly associated with PSCC tumor progression, as indicated by clinical features including the pN status, clinical stage and ENE. When correlated with survival data, Kaplan-Meier analysis showed that patients with high BCL2A1 or AIM2 expression experienced shorter CSS, and the worst survival outcomes were observed in patients with double-positive expression. Our results suggest that BCL2A1 or AIM2 staining predicts an advanced pN stage and poor survival, indicating its potential prognostic value in PSCC. Therefore, we aimed to further explore whether it could provide additional prognostic information in patients with different pN statuses.

In the pN0 subset, pN0 patients with overexpression of both BCL2A1 and AIM2 had a 5-year CSS rate of only 63.3%, which was even worse than those of the whole pN1 patient population in our cohort (5-year CSS rate: 75.0%) and patients from other centers (5-year CSS rate: 79-89%) 7, 8. However, patients with single-positive expression (5-year CSS rate: 94.9%) and double-negative expression (5-year CSS rate: 100.0%) had a good prognosis, even better than that of the whole pN0 cohort (5-year CSS rate: 90.6% in our cohort and over 85% in cohorts from other centers) 6-8. This difference in survival might be attributed to tumor heterogeneity rather than tumor progression, due to the low recurrence rate (2.8%) in pN0 patients in our previous report 44. Therefore, with the advantages of the molecular classifiers BCL2A1 and AIM2, we identified a small proportion of pN0 patients (17.6%, 19/108) at high risk of tumor progression and poor outcomes, a subset that was imperceptible by conventional pT or G stratification (Figure S3A). These classifiers are beneficial for the early detection of high-risk pN0 PSCC patients who might receive frequent follow-up and aggressive treatments.

Similarly, in the pN+ subset, the prognosis of patients with negative BCL2A1 and AIM2 expression was significantly better than that of patients with double-positive expression (5-year CSS rate: 55.1% *vs.* 24.1%). The 5-year CSS rate of low-risk double-negative pN1 patients reached up to 80.0% (66.7% in pN2), which was close to that of the whole pN0 patient population with favorable outcomes 6, 8, 10. However, the 5-year CSS rate decreased dramatically to 9.5% in double-positive pN3 patients. Our results indicate that low-risk pN+ patients with double-negative expression achieve a stable response after lymphadenectomy and a good prognosis, especially those with limited inguinal metastasis. For others with a high tumor burden, targeted therapies combined with recommended adjuvant chemotherapy might improve clinical outcomes 1. Therefore, we propose a novel molecular classifier integrating BCL2A1 and AIM2 expression with the pN status for a precise prognostic assessment and for providing additional information for clinical decision-making.

However, we found BCL2A1 but not AIM2 to be an independent prognostic indicator, and pN0 patients with single BCL2A1 expression had a worse prognosis than those with single AIM2 expression. To further demonstrate the intrinsic connection between BCL2A1 and AIM2 as well as their oncogenic functions in the progression of PSCC, convincing experiments were conducted using our newly established PSCC cell lines 20. We determined that knocking down BCL2A1 and AIM2 inhibited PSCC cell proliferation, clone formation, migration* in vitro* and tumor growth *in vivo*, supporting their critical role in tumorigenesis. Regarding the molecular mechanism, the knockdown of AIM2 significantly decreased BCL2A1 expression *via* the NF-κB pathway, suggesting that BCL2A1 is a dominant downstream oncogene that is not only regulated by AIM2 but also independently influences the progression of PSCC. Therefore, selective BCL2 inhibitors, such as venetoclax, which has been shown to be effective against hematological malignancies 45, might be an option for targeted treatment, particularly for patients with BCL2A1-positive PSCC. In addition, the MAPK pathway plays a crucial role in cell proliferation, differentiation, and apoptosis in various tumors 46. Our results demonstrated that knockdown of the BCL2A1-mediated MAPK pathway reduced the expression of the c-Myc oncogene and eventually inhibited tumorigenesis in PSCC. In conclusion, we found an unexplored mechanism by which proliferation in PSCC is promoted *via* the AIM2/NF-κB/BCL2A1/MAPK/c-Myc signaling pathway.

Furthermore, AIM2, a cytosolic double-stranded DNA sensor protein, is over expressed in B lymphocyte and is involved in innate immune signaling to regulate programmed cell death 32, 47. Thus, we try to explore whether AIM2 in PSCC cells has similar mechanisms participating in the immune response. We found that the secretion of cleaved IL-1β and IL-18 was at a low baseline level, regardless of the presence or absence of AIM2 in PSCC cells. It indicated that AIM2 might not rely on the anti-tumor inflammatory cytokines to affect immune response in PSCC cells. Besides, we explored that AIM2-silenced SCC-7 cells inhibited subcutaneous tumor growth in immune-competent mice, which implied a direct effect of AIM2 on cancer epithelial cell growth. Although the validation of the contribution of AIM2 expressed in immune/mesenchymal cells in PSCC has not been investigated, the preliminary results might provide valuable insights into the important role of tumor progression.

Our study also has some limitations. Although CGP was performed with strictly matched PSCC tissues, it was still limited by the small sample size and single transcriptomic research. In addition, we focused on the oncogenes and ignored the potential tumor suppressor genes that might be hidden among the co-downregulated genes. Second, owing to the single-center and retrospective study design, the prognostic classifiers should be further validated in other centers. Third, for high-risk pN0 patients, the optimal aggressive treatments remain unknown, and clinical trials are cautiously recommended.

## Conclusions

In this study, we propose a novel prognostic classifier that integrates molecular stratification by BCL2A1 and AIM2 expression into the pN status of PSCC that provides additional prognostic information for clinical decision-making beyond the existing clinical and pathological staging systems. Moreover, we demonstrate that the AIM2/NF-κB/BCL2A1/MAPK/c-Myc pathway is the intrinsic molecular mechanism in the progression of PSCC.

## Supplementary Material

Supplementary figures and tables.

## Figures and Tables

**Table 1:** Relationship between BCL2A1 and AIM2 expression and clinicopathological features in 220 PSCC patients

Variable	Total N	BCL2A1 staining N, %			AIM2 staining N, %		
		Low expression	High expression	*p*-value^ a^	Low expression	High expression	*p*-value^ a^
**Age**				0.128			0.812
<55	126	90 (40.9)	36 (16.4)		37 (16.8)	89 (40.5)	
≥55	94	58 (26.4)	36 (16.4)		29 (13.2)	65 (29.5)	
**pT status**				0.152^b^			**0.016^ b^**
≤pT1^c^	81	57 (25.9)	24 (10.9)		30 (13.6)	51 (23.2)	
pT2	32	23 (10.5)	9 (4.1)		14 (6.4)	18 (8.2)	
pT3	78	53 (24.1)	25 (11.4)		17 (7.7)	61 (27.7)	
pT4	10	3 (1.4)	7 (3.2)		0	10 (4.5)	
Tx	19	12 (5.5)	7 (3.2)		5 (2.3)	14 (6.4)	
**pN status**				**0.005**			**0.031**
N0	108	79 (17.7)	29 (13.2)		40 (18.2)	68 (30.9)	
N1	27	21 (9.5)	6 (2.7)		10 (4.5)	17 (7.7)	
N2	29	21 (9.5)	8 (3.6)		7 (3.2)	22 (10.0)	
N3	56	27 (12.3)	29 (13.2)		9 (4.1)	47 (21.4)	
				0.302^b^			0.288^b^
**Metastasis**							
M0	210	143 (65)	67 (30.5)		65 (29.5)	145 (65.9)	
M1	10	5 (2.3)	5 (2.3)		1 (0.5)	9 (4.1)	
**Clinical stage^d^**							
				**0.001**			**0.022**
Stage I	57	40 (18.2)	17 (7.7)		22 (10)	35 (15.9)	
Stage II	51	40 (18.2)	11 (5.0)		19 (8.6)	32 (14.5)	
Stage III	48	37 (16.8)	11 (5.0)		15 (6.8)	33 (15.0)	
Stage IV	64	31 (14.1)	33 (15.0)		10 (4.5)	54 (24.5)	
**Histology**							
				0.085			0.166
G1	122	89 (40.5)	33 (15.0)		43 (19.5)	79 (35.9)	
G2	68	43 (19.5)	25 (11.4)		16 (7.3)	52 (23.6)	
G3	30	16 (7.3)	14 (6.4)		7 (3.2)	23 (10.5)	
**ENE**				**0.003**			**0.045**
No	175	126 (57.3)	49 (22.3)		58 (26.4)	117 (53.2)	
Yes	45	22 (10.0)	23 (10.5)		8 (3.6)	37 (16.8)	

^a^Chi-square test;^ b^Fisher's exact test; ^c^included Ta, Tis and T1; ^d^clinical stage based on the AJCC Cancer Staging Manual and TNM Staging System for Penile Cancer (8th ed., 2017); ENE, extranodal extension; PSCC, penile squamous cell carcinoma.

**Table 2:** Survival analysis of different expression patterns of BCL2A1 and AIM2 in 220 PSCC patients

Variable	Total	Events^a^ (%)	5-years CSS rate (95% CI)	χ^2^	*p*-value
Total cohort of patients with PSCC	220	92 (41.8)	0.635 (0.568-0.702)		
Double-negative expression (BCL2A1^-^ AIM2^-^)	49	10 (20.4)	0.805 (0.689-0.921)	Ref.	-
Single-positive expression (BCL2A1^+^ AIM2^-^ or BCL2A1^-^ AIM2^+^)	116	41 (35.3)	0.686 (0.598-0.774)	3.128	0.077
Double-positive expression (BCL2A1^+^ AIM2^+^)^b^	55	41 (74.5)	0.389 (0.258-0.520)	25.444	0.000
Single-positive BCL2A1^+^ expression	17	8 (47.1)	0.686 (0.457-0.915)	Ref.	-
Single-positive AIM2^+^ expression	99	33 (33.3)	0.684 (0.590-0.778)	0.194	0.660
Subset of patients with pN0 PSCC	108	17 (15.7)	0.906 (0.847-0.965)		
Double-negative expression (BCL2A1^-^ AIM2^-^)	30	0	1.000	Ref.	-
Single-positive expression (BCL2A1^+^ AIM2^-^ or BCL2A1^-^ AIM2^+^)	59	6 (10.2)	0.949 (0.892-1.000)	3.151	0.076
Double-positive expression (BCL2A1^+^ AIM2^+^)^c^	19	11 (57.9)	0.633 (0.392-0.874)	23.332	0.000
Single-positive BCL2A1^+^ expression	10	4 (40.0)	0.900 (0.714-1.000)	Ref.	-
Single-positive AIM2^+^ expression	49	2 (4.1)	0.959 (0.904-1.000)	5.291	0.021
Subset of patients with pN+ PSCC^d^	93	58 (62.4)	0.400 (0.296-0.504)		
Double-negative expression (BCL2A1^-^ AIM2^-^)	14	6 (42.9)	0.551 (0.279-0.823)	Ref.	-
Single-positive expression (BCL2A1^+^ AIM2^-^ or BCL2A1^-^ AIM2^+^)	50	29 (58.0)	0.457 (0.312-0.602)	0.747	0.387
Double-positive expression (BCL2A1^+^ AIM2^+^)^e^	29	23 (79.3)	0.241 (0.086-0.396)	3.883	0.049
Single-positive BCL2A1^+^ expression	7	4 (57.1)	0.286 (0.000-0.723)	Ref.	-
Single-positive AIM2^+^ expression	43	25 (58.1)	0.470 (0.315-0.625)	0.003	0.956

^a^Number of patients who died throughout the follow-up period. ^b^Log-rank test indicated that the survival of PSCC patients with single-positive expression was better than that of patients with double-positive expression (χ^2^=20.563, *p* = 0.000). ^c^In the pN0 subset, a significant difference was also found between the single-positive and double-positive groups (χ^2^=20.700, *p* = 0.000). ^d^Nineteen Tx patients were excluded, and a total of 93 pN+ PSCC patients were analyzed in the subset. ^e^No significant difference between the single-positive and double-positive groups in the pN+ subset (χ^2^=2.569, *p* = 0.109). IHC, immunohistochemistry; Ref. reference.

**Table 3:** Univariate and multivariate analyses of 220 PSCC patients with different clinical and pathological features

Variable	Total	Univariate analysis^a^			Multivariate analysis^b^	
		Events (%)	5-year CSS rate (95% Cl)	*p*-value	Hazard ratio (95% Cl)	*p*-value
**Age**				0.073		
<55	126	52 (41.3)	0.582 (0.484-0.680)			
≥55	94	40 (42.6)	0.681 (0.587-0.775)			
**pT status^c^**				0.000		0.004
≤pT1	81	13 (16.0)	0.870 (0.796-0.944)	Reference	Reference	-
pT2	32	17 (53.1)	0.553 (0.377-0.729)	0.000	3.792 (1.728-8.321)	0.001
pT3	78	35 (44.9)	0.627 (0.515-0.739)	0.000	2.805 (1.419-5.544)	0.003
pT4	10	10 (100)	0.000	0.000	3.721 (1.385-9.998)	0.009
**Histology**				0.000		0.067
G1	122	35 (28.7)	0.787 (0.713-0.861)	Reference	Reference	-
G2	68	33 (48.5)	0.528 (0.401-0.655)	0.001	1.338 (0.740-2.418)	0.336
G3	30	24 (80.0)	0.254 (0.093-0.415)	0.000	2.263 (1.129-4.536)	0.021
**pN status^d^**				0.000		0.000
N0	108	17 (15.7)	0.906 (0.847-0.965)	Reference	Reference	-
N1	27	9 (33.3)	0.741 (0.576-0.906)	0.024	1.822 (0.734-4.525)	0.196
N2	29	17 (58.6)	0.447 (0.257-0.637)	0.000	3.559 (1.596-7.937)	0.002
N3	56	49 (87.5)	0.115 (0.019-0.211)	0.000	17.966 (6.251-51.641)	0.000
**Metastasis**				0.000		
M0	210	82 (39.0)	0.666 (0.599-0.733)		Reference	
M1	10	10 (100)	0.000		2.266 (0.954-5.385)	0.064
**Clinical stage^e^**				0.000	Excluded^f^	
Stage I	57	5 (8.8)	0.943 (0.880-1.000)	Reference		
Stage II	51	10 (19.6)	0.937 (0.868-1.000)	0.051		
Stage III	48	21 (43.7)	0.634 (0.495-0.773)	0.000		
Stage IV	64	56 (87.5)	0.102 (0.016-0.188)	0.000		
**ENE**				0.000	Excluded^f^	
No	175	54 (30.9)	0.762 (0.697-0.827)			
Yes	45	38 (84.4)	0.118 (0.004-0.232)			
**BCL2A1 stain**				0.000		
Low	148	43 (29.1)	0.724 (0.650-0.798)		Reference	
High	72	49 (68.1)	0.458 (0.340-0.576)		1.691 (1.014-2.820)	0.044
**AIM2 stain**				0.004		
Low	66	18 (27.3)	0.773 (0.667-0.879)		Reference	
High	154	74 (48.1)	0.576 (0.496-0.656)		1.346 (0.720-2.518)	0.352

^a^Log-rank test; ^b^Cox regression model (Tx patients excluded, n = 201); ^c^Tx patients were excluded (n = 201), and the stratified analysis revealed no significant difference between pT2/pT3 (χ^2^ = 0.317; p = 0.573); ^d^Stratified analysis indicated no significant differences between pN1/pN2 (χ^2^ = 2.927; p = 0.087); ^e^Stratified analysis indicated no significant differences between stages I/II (χ^2^ = 3.793; p = 0.051). ^f^Clinical stage and ENE were excluded from the Cox regression model because they could be represented by TNM stage. CSS, cancer-specific survival.

## Abbreviations

AFP: alpha-fetoprotein
β-hCG: beta-human chorionic gonadotropin
CGP: comprehensive genomic profiling
CI: confidence interval
CSS: cancer-specific survival
CCK-8: cell counting kit-8
DMEM: Dulbecco's Modified Eagle's Medium
ELISA: enzyme-linked immunosorbent assay
ENE: extranodal extension
ER: estrogen receptor
FC: fold change
HCPS: high-content proliferation screening
Her2: human epidermal growth factor receptor 2
HR: hazard ratio
IHC: immunohistochemistry
LDH: lactate dehydrogenase
MAPK: mitogen-activated protein kinase
mRNA: messenger RNA
NF-κB: nuclear factor κB
pN0: pathological node-negative
pN+: pathological node-positive
PSCC: penile squamous cell carcinoma
PR: progesterone receptor
qPCR: quantitative polymerase chain reaction
shRNA: short hairpin RNA
SYSUCC: Sun Yat-sen University Cancer Center
WB: Western blot
