# Supplementary material for: Molecular stratification by BCL2A1 and AIM2 provides additional prognostic value in penile squamous cell carcinoma
Source: Theranostics. 2021 Jan 1;11(3):1364–76. doi: 10.7150/thno.51725 (PMC7738875; doi:10.7150/thno.51725)
Supplement: Supplementary file 1 — Supplementary figures and tables. [file thnov11p1364s1.pdf]

Molecular stratification by BCL2A1 and AIM2 provides additional prognostic value in penile squamous cell carcinoma

Supplementary Figures

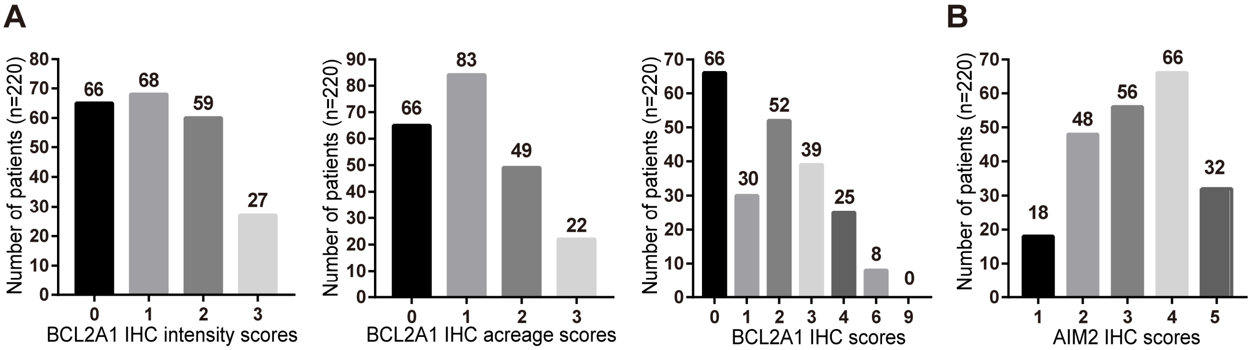

Figure S1. Frequency of BCL2A1 and AIM2 IHC staining scores in 220 PSCC patients.

(A) The IHC score for BCL2A1 was multiplied by the intensity score and the average score of patients with PSCC was reported. The frequency of each score is displayed. The cut-off value of the BCL2A1 IHC score was 2 points, and 3-9 points was regarded as BCL2A1 overexpression in patients with PSCC. (B) The IHC scores for AIM2 expression depended on the staining intensity. The patients in each group are shown; 2 points was the cut-off value, and 3-5 points was regarded as high AIM2 expression. IHC, immunohistochemistry; PSCC, penile squamous cell carcinoma.

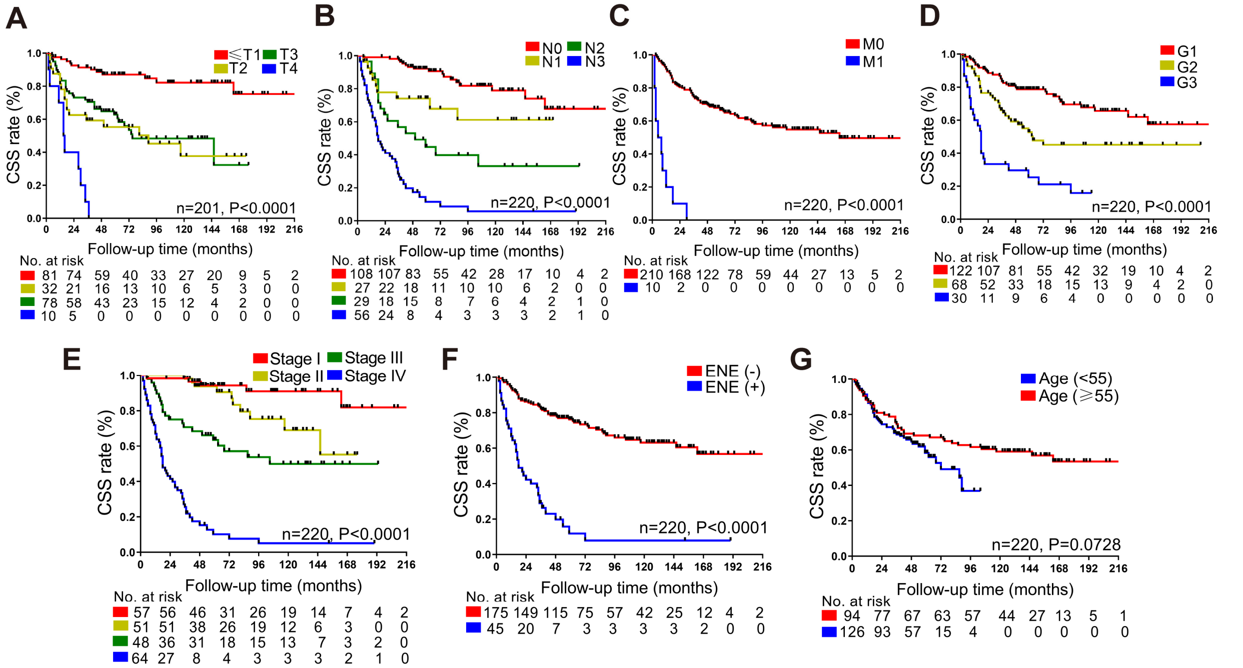

Figure S2. Relationships between clinical and pathological features and survival in 220 PSCC patients.

Kaplan-Meier survival curves revealed that (A) the T grade, (B) N grade, (C) metastasis, (D) pathological grade, (E) clinical stage, and (F) ENE were associated with CSS in 220 patients with PSCC, but not (G) age. (A) The hierarchical analysis did not reveal a difference between T2 and T3 ($\chi^2 = 0.321$, p = 0.570), while a significant difference was observed between the remaining groups (≤ T1 included Ta, Tis and T1). The clinical and pathological features were based on the TNM Staging System for Penile Cancer (8th ed., 2017). ENE, extranodal extension; PSCC, penile squamous cell carcinoma.

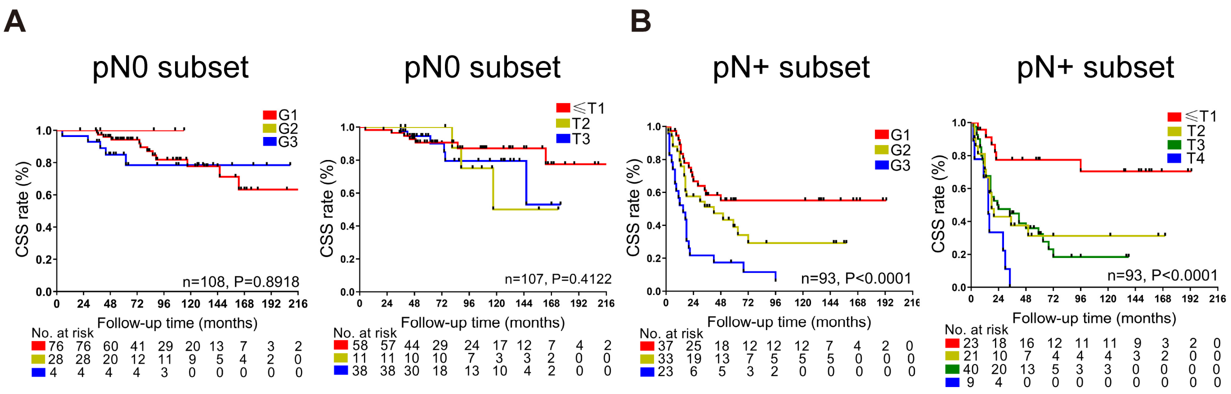

Figure S3. Survival of patients with PSCC of different pN statuses based on stratification according to the pT grade and G grade.

(A) In the pN0 subset, different T grades and G grades were not associated with CSS (one patient with T4N0M0 who died of cancer at 37 months was excluded from the T grade survival curve).

(B) In the pN+ subset (n = 93, Tx patients were excluded), the T grade and G grade were associated with disease-specific survival (DSS). The TNM grade and pathological grade were based on the TNM Staging System for Penile Cancer (8th ed., 2017). IHC, immunohistochemistry; PSCC, penile squamous cell carcinoma.

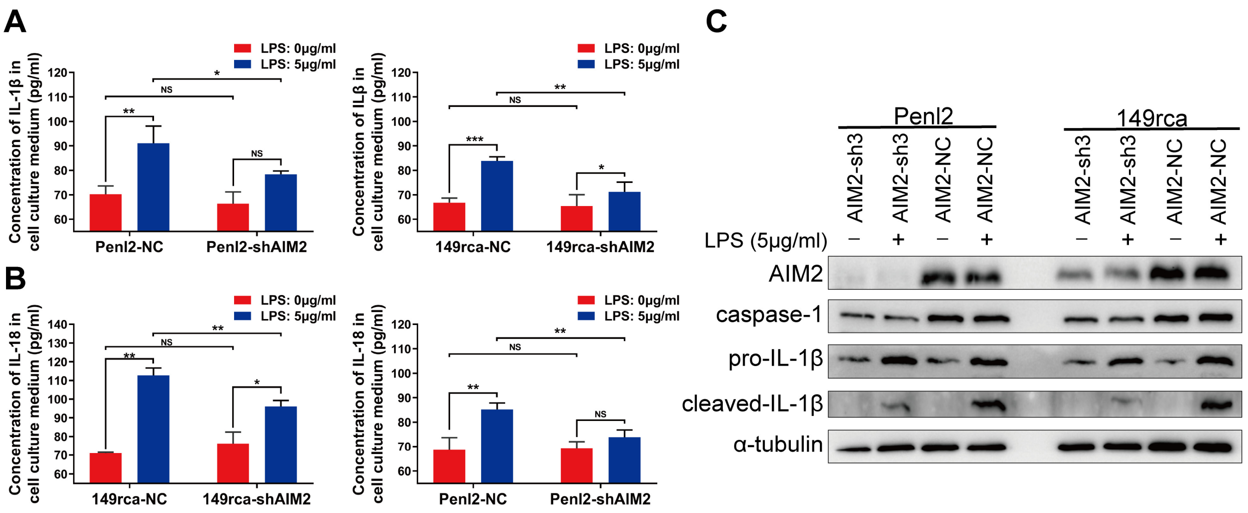

Figure S4. The relation between AIM2 expression and the release of inflammatory cytokines in PSCC cells.

(A) In the normal tumor microenvironment (without exogenous LPS stimulation), an extremely low level of the activated inflammatory cytokine IL-1β (cleaved-IL-1β) was detected in Penl2 and 149rca PSCC cells. Knockdown of AIM2 expression in PSCC cells reduced Caspase-1 levels, but did not affect the levels of pro-IL-1β and mature IL-1β. When LPS was added, the AIM2 inflammasome was significantly activated and increased the levels of pro-IL-1β and mature IL-1β, which were particularly increased, in the NC cells compared to the AIM2-silenced cells. (B and C) Knockdown of AIM2 expression in Penl2 and 149rca cells did not alter the release of IL-1β and IL-18 into the culture medium in the absence of LPS stimulation, as determined using ELISAs. Additionally, exogenous LPS increased the secretion of IL-1β and IL-18 by PSCC cells and the concentrations of IL-1β and IL-18 were higher in the NC groups than in the AIM2-silenced groups. *p < 0.05, **p < 0.01, ***p < 0.001. LPS, lipopolysaccharide; IL, interleukin; ELISA, enzyme-linked immunosorbent; NC, negative control; NS, not significant.

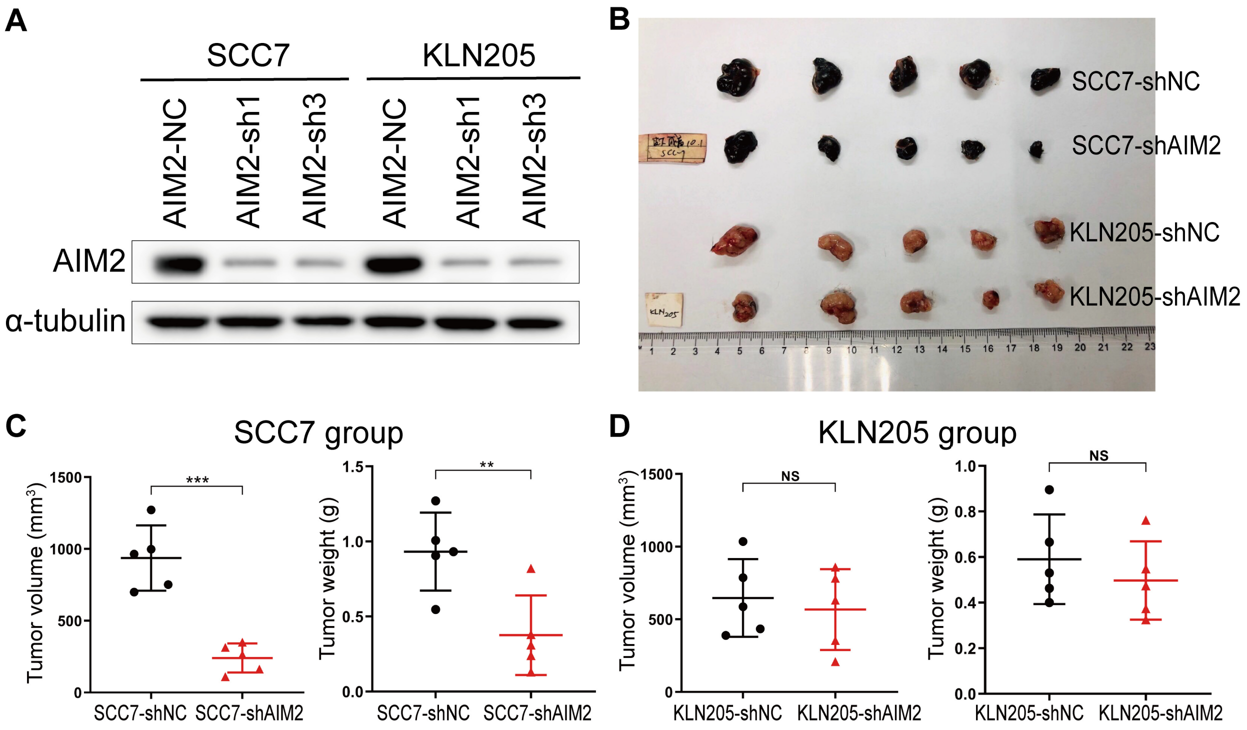

Figure S5. Knockdown of AIM2 in SCC-7 cells inhibits tumor growth in immune-competent mice

(A) Knockdown efficiency of the expression of the AIM2 protein in the mouse head and neck squamous cell carcinoma (SCC-7) and mouse lung squamous cell carcinoma (KLN205) cell lines. (B-D) Knockdown of AIM2 in SCC-7 cells significantly inhibited tumor growth *in vivo*, while knockdown of AIM2 expression in KLN205 cells did not exert a significant effect on tumor growth.

Supplementary Tables

Table S1. Clinicopathological features of eight patients with pN+ PSCC included in the subsequent CGP analysis.

Patient No.	Age	Surgical pattern	TNM stage	Pathological stage	Lymphovascular /perineural invasion	ILNM	PLNM	ENE
1	44	P+IID+PID	T3N3M0	G2	No	Right	No	Yes
2	49	P+IID+PID	T1aN3M0	G2	No	Bilateral	Yes	Yes
3	76	P+IID+PID	T2N2M0	G2	No	Bilateral	No	No
4	71	P+IID+PID	T3N3M0	G2	Yes	Left	No	Yes
5	65	P+IID+PID	T1aN3M0	G1	No	Right	No	Yes
6	54	P+IID	T2N1M0	G1	No	Left	Uk	No
7	78	P+IID+PID	T4N2M0	G2	Yes	Bilateral	No	No
8	57	P+IID	T2N2M0	G2	No	Bilateral	Uk	No

Eight patients were newly diagnosed with pN+ PSCC at SYSUCC between February 2016 and November 2016 and had not received any previous treatment. Fresh frozen samples were retrieved in pairs, and CGP was immediately performed after the pathology was confirmed. The TNM stage and pathological stage were based on the TNM Staging System for Penile Cancer (8th ed., 2017). PSCC, penile squamous cell carcinoma; CGP, comprehensive genomic profiling; SYSUCC, Sun Yat-sen University Cancer Center; P, penectomy; IID, radical inguinal lymphadenectomy; PID, radical pelvic lymphadenectomy; ILNM, inguinal lymph node metastasis; PLNM, pelvic lymph node metastasis; ENE, extranodal extension.
